# Cases of Infantile Paralysis—Paraplegia by Rheumatic Metastasis, Ending in Hemiplegia and Recovery, and Cerebral Paralysis

**Published:** 1878-02

**Authors:** J. J. Caldwell

**Affiliations:** Baltimore, Md.


### CASES OF INFANTILE PARALYSIS—PARAPLEGIA BY RHEUMATIC METASTASIS ENDING IN HEMIPLEGIA AND RECOVERY AND CEREBRAL PARALYSIS.

BY J. J. CALDWELL, M. D., BALTIMORE, MD.

*Journal of Nervous and Mental Diseases, April, 1876.*

Johnny S., 2½ years old, was recommended to our office, by his physician, Dr. Correl of Baltimore, January 23d, 1875. History: Complete paralysis of the lower extremities, with tendency to double talipes varus; partial loss of sensation with lowering of temperature; and partial atropy of the affected parts. The electro-muscular contractility, under the faradic current, poor; under the galvanic current far better, so much so that we could offer a fair prognosis. The mother informed us that during the heat of the previous summer, the child, whilst cutting teeth, suffered great prostation. In the midst of this illness it was seized with a slight convulsion, and for several days following was comatose, but gradually improved from this condition, when she discovered that the child was paralyzed, as mentioned.

My mode of procedure was warm baths to the lower extremities, and daily lubrications as follows:

℞ Phosphori, Solidi grs. viii.
Olei Olivi Kalidæ ℥ iv.
Ft. Sol.

S. To be applied to the affected parts morning and evening, after thorough massage. Made application of galvanism as follows: A large and dampened electrode (positive) was applied to the sacral plexus, while the hand of the operator was applied as negative to every atonic and atrophied muscle; application on alternate days, of from fifteen to thirty minutes duration.

Internally we exhibited the following:

℞ Strychnie Sulph. gr. i.
Acidi Phosphor, Dil. ℥ i.

S. Five to ten drops in sweetened water, twice a day.

The foregoing treatment was continued from January 23d, 1875, to May 14th, 1875. Faradism was alternated with the galvanic current during the last half of May, the difference in their essential qualities, being, that while galvanism, by its action on the vaso-motor, produces extra nutrition to the parts as is demonstrated by the ready congestion, faradism seems to bend its especial action to muscular tonicity and elasticity, especially after the muscles have received this quality to a moderate degree from the galvanic current.

The result in the treatment of this child's case was very flattering, for when he left me last summer, to be taken to the country, he could walk around the room, from chair to chair, and stand alone, though I regarded it as a typical case of infantile paralysis.

Remarks.—The cause of this disease remains an obscure and mooted question. Though it has been most frequently observed during the age of dentition, it occasionally, very suddenly appears after exposure to low temperatures of damp and shady places, by inadvertence of the nurse or parents, especially if the child's perspiration is suddenly checked. It is, also, frequently a sequel of various diseases common to infancy, such as the contagious and eruptive disorders, and summer complaints or fluxes.

Morbid Anatomy.—Under this heading one might readily inquire into the relation between vascular paresis and fatty, or morbid cell development, from various depressing or surrounding malign influences. Certainly a change of action would give us a change of results.

Says Hammond, "the morbid anatomy of organic infantile paralysis is to be studied in the spinal cord, the nerves and the muscles."

As regards the muscles there has been a tolerable accord among observers; but there has been no approach to uniformity, relative to the state of the spinal cord and nerves. The general opinion has been that there is no appreciable alteration. The above author says he found a cicatrix, with a small clot. Paralysis in this case was in the left lower extremity, and of four years duration. The lesion existed in the lower part of the dorsal cord, in the left anterior column. Dr. Lockhart Clark has given a great impetus to the studies of the morbid anatomy of these parts, especially in relation to infantile paralysis.

In his microscopic observation, in one case, the spinal cord was found to be affected from the cervical to the lumbar enlargement. The alteration was chiefly in the gray matter, and especially in the anterior cornua. These were atrophied and distorted; and the cells had disappeared to a very great extent. The posterior cornua was affected, though in much less extent. The anterior root of the nerve, proceeding from the diseased portion of the cord, was atrophied. The paralyzed muscle had undergone fatty transformation, and the fibrilli had, to a great extent, disappeared.

*Rheumatic paralysis of the Cord, producing Paraplegia.*—January 3d, 1875, I was called by Dr. A. W. Dodge, of Baltimore, in consultation to see Mrs. Barbara C., aged fifty, who had suffered some weeks previous with articular rheumatism. We found her in bed suffering with paraplegia of the lower extremities, owing to a rheumatic metastasis to the membranes of the lower portion of the spinal cord. The severe rheumatic pains of the joints had entirely disappeared, suddenly producing pain in the lower portion of the cord, with effusion and total paralysis, an œdematous condition of the feet and legs.

Upon an electrical examination I found the superior extremities and body in a normal condition, until the seat of the effusion was reached, (the middle dorsal vetebræ). From that point to her toes there was a loss of sensation, motion and temperature. The line of demarcation was well defined, as indicated by the strong faradization of the lower extremities, producing little or no apparent effect on, and below, the seat of injury, whilst above this point, it was unbearable. Percussion of the spine furnished the same diagnosis.

We ordered iodide of potash and nux vomica in moderate and increasing doses; also the local application of faradism, daily with the best results. This treatment was continued until the middle of February, when the patient was able to move about, with a freedom from her rheumatic and spinal difficulty. Thus she continued to improve until April 25th, when I was recalled and found her prostrated with an attack of hemiplegia, which seized her whilst in a stooping posture, she being a heavy, plethoric woman.

This time her whole right side was affected, with the tongue and eye divergent. I placed her on a mild diet and administered iodide of potash, in large doses, which I continued for a few weeks, and then resorted to a faradic current, locally and generally. This has been continued for about a year with a gradual progress to convalescence. All the acute symptoms having been subdued, she is now able to sit up and walk around her room, to a limited extent.

Remarks.—The anomalous features of this case were, that she should have been first seized with rheumatic paraplegia, involving the lower half of the cord, with effusion, ending in absorption and recovery, and that a few months thereafter she should have been seized with brain paralysis.

Rheumatic paralysis is spoken of by Althaus and other writers as being a frequent occurrence with hunters, sportsmen, and others who are subject to exposure, especially in damp seasons or climates.

Again this case is rare, as I have yet to find a history of a similar affection of the cord, especially by rheumatic metastasis; though Hammond and others speak of its effects upon the membranes of the cerebrum.

Duchenne has found that in this form of palsy "the electro-muscular contractility is normal, while the sensation excited by the electro-muscular contraction may be stronger in the suffering side than in the healthy parts." Althaus says that this is true for recent cases, but in those of long standing he had almost invariably found farado-muscular excitability impaired.

*Cerebral Paralysis.*—Mr. C., a gentleman of fifty-five years of age, placed himself under my care, October, 1874, suffering from hemiplegia of the right side, with the following history:

About a year and a half since he was seized, one summer evening, after undergoing great mental and physical fatigue with the attack,—suddenly, and without forewarning. When aroused from a profound slumber it was discovered that he could not arise from his couch. After rest and treatment for a few months he suffered a second seizure, for which he underwent an alterative treatment and mild regimen for about a year.

On the 20th, of October, (1874) I made my first examination, and found him a well preserved man, weighing about one hundred and fifty pounds; eye and tongue normal, but speech greatly impeded, (partial aphasia;) deglutition somewhat impaired, with the arm and leg of the affected side partially paralyzed.

The nutrition and sensation were alike preserved on both sides, as also were the temperature, muscular tonicity, and contractility. In this case there seemed only to be a lesion of the anterior convolutitions, producing partial aphasia from the loss of co-ordination, digestion and the general functions of the organs were unimpaired, and there was no insomnia. He could only walk short distances and by assistance alone. Having lost the power of volition over the muscles of the affected side he was unable even to transcribe his thoughts on paper.

Regarding this particularly as a local trouble I directed my treatment to the parts affected, viz: gentle applications or constant galvanic currents to the brain and sympathetic, with the exhibition of iron, strychnine, and phosphorus, in small doses. This treatment has been continued for four months, with the following results: When not excited, the patient is able to articulate quite distinctly for five or ten minutes at a time. He is now able to convey his food to his mouth with his right hand, write a few words, and stand alone.

